# Microfluidic transection injury and high-shear thrombus formation demonstrate increased hemostatic efficacy of cold-stored platelets and *in vitro* resuscitation in induced coagulopathy models

**DOI:** 10.3389/fbioe.2025.1568113

**Published:** 2025-05-12

**Authors:** Emily P. Mihalko, Refael Munitz, Devin M. Dishong, Skye Clayton, Susan M. Shea

**Affiliations:** ^1^ Trauma and Transfusion Medicine Research Center, Department of Surgery, University of Pittsburgh, Pittsburgh, PA, United States; ^2^ Department of Bioengineering, University of Pittsburgh, Pittsburgh, PA, United States

**Keywords:** platelets, resuscitation, coagulopathy, hemostasis, cold-stored platelets

## Abstract

Hemostatic resuscitation is an essential aspect of treating traumatic bleeding. Trauma-induced coagulopathy is a multifactorial disorder that can lead to increased transfusion requirements. However, little is known about the interplay between coagulopathies and stored blood products used for hemostatic resuscitation, which themselves acquire dysfunction in the form of a storage lesion. Physiologically relevant models can aid in the study of trauma and hemostatic resuscitation by incorporating important aspects such as biological surfaces and flow regimes that mimic injury. This study aims to evaluate the contribution of platelet products under varying storage conditions in coagulopathic states. This study utilized microfluidic platforms of high shear, a flow regime relevant to injury, including a stenotic straight channel and a severe transected vessel injury device. Apheresis platelet products were collected from healthy volunteers, stored at room temperature (RT) or cold-stored (CS) (1°C–6°C), and tested for product cell count and intrinsic product function in a high-shear stenotic microfluidic device across storage days (D2, D5, and D7 for RT; D2, D5, D7, D14, and D21 for CS). Hemostatic resuscitation efficacy of products was assessed using induced coagulopathy models of dilution and thrombocytopenia (TP). *In vitro* hemostatic resuscitation was assessed in both the stenotic straight channel for kinetic platelet contributions and the transected-vessel injury device, using blood loss and clot composition as endpoints. CS products conserved inherent function despite decreasing platelet counts through storage D7. When mixed with coagulopathic blood, D2 RT products did not show hemostatic benefit in the dilutional coagulopathy (DC) model. However, both D2 RT and CS showed hemostatic benefits in the thrombocytopenia model. CS products (D5 and D7) also showed an enhanced ability to recruit recipient platelets in the thrombocytopenia model compared to RT. Overall, this study highlights disparate responses associated with product storage duration and temperature, indicating the need to further evaluate hemostatic resuscitation efficacy under flow in pathologically relevant models to guide transfusion practices.

## 1 Introduction

Hemorrhagic shock due to excessive blood loss is a leading cause of death in trauma patients, accounting for up to 1.5 million deaths worldwide each year (Lozano et al., 2012). The traumatic injury response is complex [reviewed by Moore et al. (2021) and Vulliamy et al. (2021)], with both adaptive and maladaptive aspects. Consumption of clotting factors, hypocoagulability, hyperfibrinolysis, and iatrogenic resuscitation-associated coagulopathies (e.g., dilution) can perpetuate a lack of clotting response, leading to trauma-induced coagulopathy (Kushimoto et al., 2017; Moore et al., 2021). Additionally, platelet dysfunction after injury is a key component of trauma-induced coagulopathy (Kutcher et al., 2012; Vulliamy et al., 2020; Sloos et al., 2022).

Early hemostatic resuscitation following trauma is associated with improved survival (Sperry et al., 2018; Torres et al., 2024), and specifically, early platelet transfusion is critical (Gunter et al., 2008; Holcomb et al., 2008). However, stored blood components acquire a storage lesion, which results in products with their own unique coagulopathy and manifests in reduced hemostatic function as storage progresses (Cap, 2017; Shea et al., 2019). It remains to be studied how blood products, along with their acquired dysfunction during storage, interact with patient hemostatic function in the context of trauma-induced coagulopathy.

*In vitro* and *ex vivo* models that incorporate critical aspects of hemostasis are especially valuable for the functional evaluation of trauma-induced coagulopathies and blood product function. Fluid dynamics play a critical role in the mechanisms underlying hemostasis and thrombosis. Therefore, this study evaluated a severe injury model with a transected vascular channel for physiological relevance to the severely injured bleeding patient (Yakusheva et al., 2022). High-shear flow was evaluated throughout this study as cellular functions under high shear are of critical importance for hemostasis, especially for platelets undergoing shear-induced platelet activation and interaction with the von Willebrand factor (Andrews et al., 1997; Shankaran et al., 2003; Colace and Diamond, 2013; Zhang et al., 2022).

Cold-stored (CS) platelet products have logistical benefits compared to storage at room temperature (RT), including increased shelf life (Shea et al., 2024). CS platelets also may have better hemostatic function than RT products (Reddoch et al., 2014; Reddoch-Cardenas et al., 2019; D’Alessandro et al., 2020; Shea et al., 2022; George et al., 2023). The platelet storage lesion is modulated by temperature [reviewed by Hegde et al. (2018), Ng et al. (2018), and Shea et al. (2019)]. In RT-stored platelets, a progressive reduction in mitochondrial function occurs over time, along with the release of extracellular vesicles, granules, and other soluble mediators (e.g., CD40L) and increased CD62P expression (Shea et al., 2019). CS platelets have improved bioenergetics (Johnson et al., 2016; D’Alessandro et al., 2020; George et al., 2023; Shea et al., 2024); however, cold stimuli cause irreversible changes to the platelet cytoskeleton (Winokur and Hartwig, 1995; Hoffmeister et al., 2001; Hegde et al., 2024), resulting in increased surface expression of CD62P, GMP140, and CD40L and increased granule mobilization (Triulzi et al., 1992; Wood et al., 2016; Shea et al., 2019, 2024). Although CS results in the aforementioned improved *in vitro* hemostatic function and longer shelf life, the impact of the CS phenotype *in vivo* in hemostatic resuscitation remains unclear.

Simulated hemostatic resuscitation using platelet products stored under different durations and temperatures may help improve our understanding, thereby informing current transfusion practices and supporting the development of improved therapeutic approaches and novel therapies. This study evaluated platelet function over product storage duration and temperature in the context of trauma-induced coagulopathy. We hypothesized that platelet products used in clinical transfusion would rescue hemostatic function in models of trauma-induced coagulopathy, including increased platelet deposition and reduced blood loss, and CS platelets would have sustained efficacy over storage compared to RT platelets.

## 2 Materials and methods

The studies involving human participants were reviewed and approved by the University of Pittsburgh Human Research Protection Office (IRB 21110093 and 21100141). Participants provided their written informed consent to participate. Inclusion and exclusion criteria for apheresis platelet and whole blood (WB) samples are listed in supplementary materials.

### 2.1 Platelet unit collection

**FIGURE 1:**
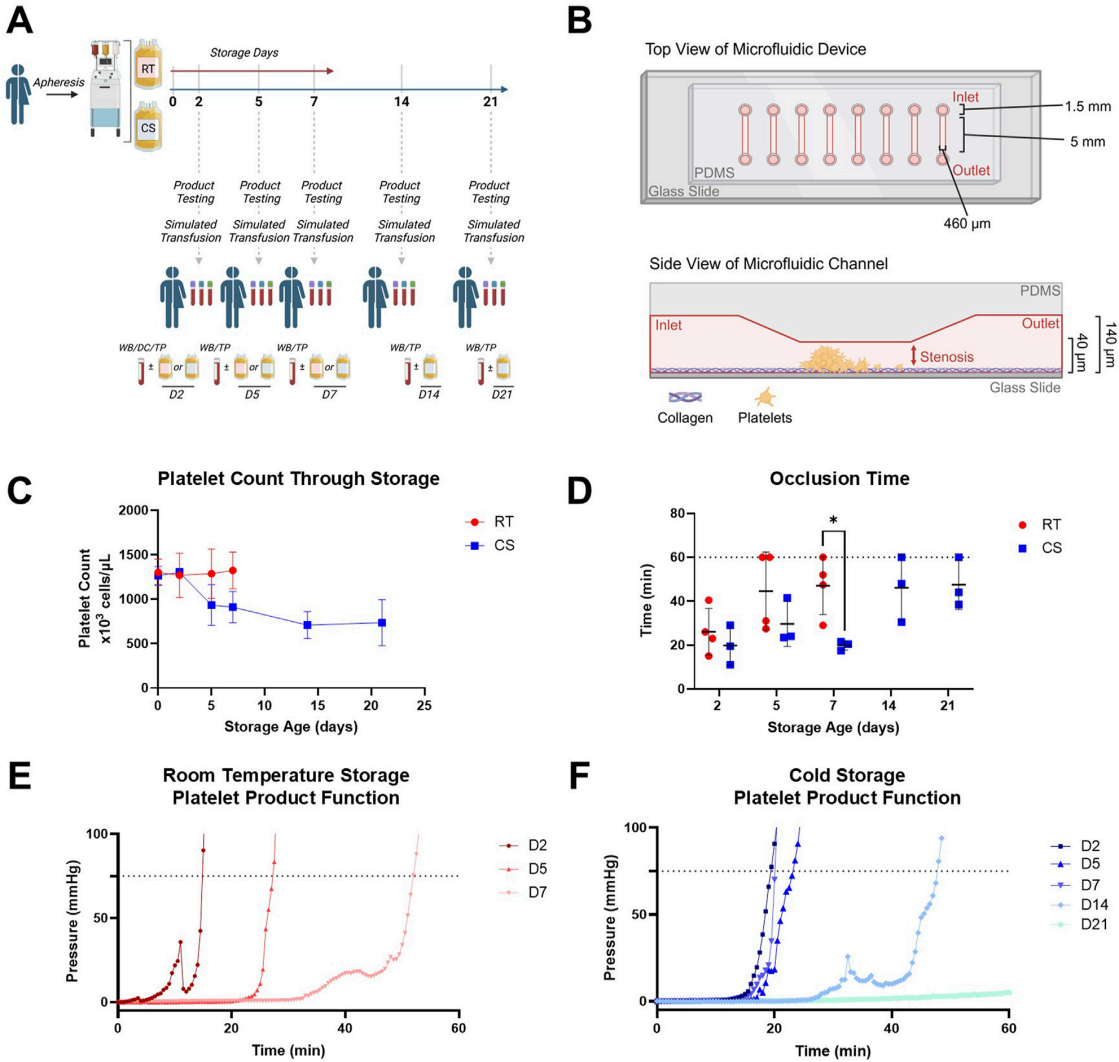
Study schematic and platelet product characterization including functional data in a stenotic microfluidic device. **(A)** Schematic representation of study design with timepoints for RT and CS apheresis platelet product testing and simulated transfusion used coagulopathy models of DC or TP with paired and unmanipulated whole blood controls (WB). **(B)** Schematic representation of the stenotic straight channel microfluidic device with assembly, dimensions, and coating used to assess inherent product platelet function with growing thrombus in the stenotic region; increasing pressure monitored inline and upstream of the device. **(C)** Platelet counts in RT (red circles) and CS (blue squares) platelet products over their storage duration. **(D)** Occlusion time based on a pressure threshold of 75 mmHg from the stenotic straight channel device in both RT (red circles) and CS (blue squares) platelet products over their storage duration. Dotted line represents an assay cap of 60 min. **(E)** Representative pressure curves from RT and **(F)** CS products are shown. Data are presented as mean ± standard deviation or individual values with bars representing mean ± standard deviation. Occlusion time data were analyzed via multiple unpaired t-tests. *p < 0.05.

All platelet products were collected from healthy donor volunteers via apheresis, following the standards of the Association for the Advancement of Blood and Biotherapies (AABB) and US Food and Drug Administration (FDA), and suspended in anticoagulant citrate dextrose solution A (ACD-A) and plasma, using the Trima Accel automated blood collection system. Platelet units were randomly assigned to CS or RT storage. RT platelets were stored with gentle agitation. CS platelets were stored between 1°C and 6°C without agitation. The agitation of RT platelets and no agitation of CS platelets are consistent with current blood banking practices in the US, and we have previously demonstrated that agitation makes no difference in RT or CS storage with respect to functionality (Shea et al., 2022). Aseptic sampling was conducted on days D2, D5, and D7 for RT products and D2, D5, D7, D14, and D21 for CS products for subsequent testing (Figure 1A). A complete blood count (ADVIA 2120i Hematology System, Siemens) and high-shear stenotic microfluidic assays on products alone were performed prior to *in vitro* resuscitation experiments. A total of eight biological replicate RT units were collected and tested on D2 with dilutional coagulopathy (DC). For extended storage studies, a total of four biological replicate RT units and three biological replicate CS units were collected and tested with thrombocytopenia (TP) coagulopathy. Sampling of each product over the storage period led to a sample size of 12 for RT products and 15 for CS products.

### 2.2 Whole blood collection for simulated *in vitro* hemostatic resuscitation with platelet products

Whole blood was collected from healthy volunteer donors via venipuncture into heparin, citrate, and EDTA vacutainers. Blood samples from these donors were used as a “recipient” for modeling transfusion through *in vitro* mixing with platelet products. Complete blood counts were performed on the healthy sample using the EDTA aliquot prior to any further processing to confirm healthy volunteer donors had normal platelet counts. The high-shear stenotic microfluidic assay, also used on product alone, and a microfluidic transection injury assay were performed on mixed samples using citrated and heparinized whole blood respectively. Paired WB, induced coagulopathy [DC or TP], and induced coagulopathy mixed with platelet product samples were run on D2, D5, and D7 for RT products and D2, D5, D7, D14, and D21 for CS products. Dose responses of the coagulopathy models were conducted and assessed for platelet count, platelet aggregometry, and rotational thromboelastometry (ROTEM), according to the manufacturer’s instructions for extrinsic pathway activation (EXTEM).

### 2.3 Stenotic microfluidic assay for the inherent platelet product function

A stenotic microfluidic device was fabricated as previously described (Mihalko et al., 2024). Channel height and width were measured on the resulting PDMS cast (Bruker DektakXT Surface Profiler and Zeiss Zen). Channel dimensions were 40 ± 3 μm in height and 462 ± 3 μm in width at the stenotic region and 137 ± 4 μm in height and 462 ± 3 μm in width at the inlet and outlet. The device was coated with collagen using a 5:1 volumetric ratio of normal saline and collagen (Chrono-par collagen reagent, Chrono-log Corporation) for 1 hour at RT. It was then rinsed with phosphate-buffered saline, incubated with 5% bovine serum albumin for 30 min, rinsed again with phosphate-buffered saline, and stored in a humid environment prior to use. Platelet products were infused through the channel at high shear rates (3,500 s^-1^) using a syringe pump (Harvard Apparatus, PHD Ultra), with upstream calcium addition using a second syringe pump (10 mM final concentration). Time to occlusion was determined using an upstream pressure monitor, with a 75 mmHg pressure increase set as the threshold for occlusion.

### 2.4 Transected injury microfluidic device fabrication

A transected vessel injury microfluidic device mold was fabricated using photolithography with a channel height of 100 μm throughout and a channel width of 300 μm at the vessel injury region, 1800 μm at the inlet, and 2000 μm at the outlet (i.e., extravascular region). An EDTA channel was positioned prior to the device outlet to quench downstream coagulation via a constant flow of EDTA (50 mM) using a syringe pump (8 μL/min) to ensure legitimate occlusion is captured at the site of interest (Berry et al., 2021). The device was coated with collagen and tissue factor using a 2:2:1 volumetric ratio of normal saline, collagen, and tissue factor (Dade Innovin, Siemens) for 1 hour at RT, rinsed with phosphate-buffered saline, then incubated with 5% bovine serum albumin for 30 min, rinsed again with phosphate-buffered saline, and stored in a humid environment prior to use.

### 2.5 Functional assessment of simulated hemostatic resuscitation

For stenotic microfluidic experiments, platelet product aliquots were incubated with Fc receptor block (Biolegend; Human TruStain FcX; 422302; 1:600) and fluorescently tagged CD41 antibody (Janelia Fluor^®^ 646) (Novus Biologicals; NB100-2614JF646; 1:600). Citrated whole blood was incubated with Fc receptor block (1:600) and a distinct fluorescently tagged CD41 antibody (Janelia Fluor^®^ 549) (Novus Biologicals; NB100-2614JF549; 1:600). DC was induced by combining whole blood with sterile normal saline in a 2:3 volumetric ratio. TP samples were induced via centrifugation. In brief, whole blood was centrifuged at 100 × *g* for 10 min with no brake. The platelet-rich plasma (PRP) layer was then removed and further centrifuged at 800 × *g* for 10 min with no brake. Platelet-poor plasma (PPP) obtained from the second spin was mixed with red blood cells saved from the first spin to create a thrombocytopenic blood sample. The platelet product was mixed with coagulopathic blood in a 1:5 volumetric ratio, equivalent to the transfusion of four platelet units. A complete blood count was performed for TP samples and TP samples mixed with platelet products. All blood samples were perfused under constant flow using a withdrawal syringe pump at an initial wall shear rate of 3,500 s^-1^ at the stenosis throat, consistent with high-shear injury (Yakusheva et al., 2022). Kinetic immunofluorescent images were captured using an Axio Observer inverted microscope (Zeiss) at 10× magnification as clot formation progressed (0.29 frames/second) under room temperature conditions. MATLAB software was used to quantify platelet deposition. Mean fluorescent intensity (MFI) was determined for each image frame and each color channel to quantify the amount of corresponding fluorescently labeled platelets deposited in the channel. MFI curves generated demonstrate platelet kinetics over the length of the experiment.

For the microfluidic experiment modeling vessel transection hemostatic capacity, platelet product aliquots were incubated with Fc receptor block (1:600) and fluorescently tagged CD41 antibody (Janelia Fluor^®^ 646) (Novus Biologicals; NB100-2614JF646; 1:600). Heparinized whole blood was incubated with Fc receptor block and a distinct fluorescently tagged CD41 antibody (Janelia Fluor^®^ 549) (Novus Biologicals; NB100-2614JF549; 1:1,200). Coagulopathies were induced as mentioned above. Platelet products were mixed with coagulopathic blood in a 1:5 volumetric ratio, simulating a volumetric transfusion of approximately four platelet units in an adult. Blood samples were perfused under constant pressure (20 mmHg) via gravity, resulting in a pathologically high shear regime at the point of transection (∼9,500 s^-1^), again consistent with high shear conditions associated with injury (Yakusheva et al., 2022). Device outflow was monitored using a scale and LabVIEW software, yielding a blood loss endpoint. Immunofluorescent images were captured at 10× magnification using an Axio Observer inverted microscope (Zeiss) (0.1 frames/second) at room temperature conditions. ImageJ software was used for platelet area quantification.

### 2.6 Statistical analysis

All statistical analysis was performed using GraphPad Prism 10 (GraphPad, San Diego, CA). Data were analyzed using an unpaired t-test for inherent product function, a paired t-test for coagulopathy blood loss, and mixed-effects analysis or one-way analysis of variance (ANOVA) with correction for multiple comparisons using an Tukey’s post hoc test with a 95% confidence interval for hemostatic resuscitation mixing studies. An unpaired t-test was conducted comparing early and late storage age blood loss. To identify outliers in platelet area data, the ROUT method—combining robust regression and outlier removal—was used, with the false discovery rate (Q) set at 1%. One outlier was identified and removed prior to analysis using an unpaired t-test. MFI fold-change curves are presented as mean ± standard error of the mean. All other data are presented as mean ± standard deviation. Data from product assessment and *in vitro* hemostatic resuscitation endpoints in the TP model were subjected to a Pearson’s correlation coefficient analysis.

## 3 Results

### 3.1 Platelet product cell count and function over storage

Platelet counts from RT and CS platelet products are shown in Figure 1C. RT products had consistent platelet counts over storage, whereas CS products decreased platelet count following D2. Intrinsic product function evaluated in the straight stenotic microfluidic channel (Figure 1B) showed a significant reduction in occlusion time (i.e., faster clot formation) with CS products compared to that with RT products at D7 (Figure 1D). Representative pressure curves from intrinsic product function testing (Figure 1E,F) illustrated conserved hemostatic function from CS products at D2, D5, and D7, which subsequently decreased at D14 and D21. Comparatively, RT products precipitously decreased in hemostatic activity over storage.

### 3.2 *In vitro* hemostatic resuscitation in a stenotic microfluidic device

Dose responses of dilutional coagulopathy revealed significantly impaired clotting parameters at a 2:3 volumetric ratio (i.e. 40% whole blood) on ROTEM and reduced platelet aggregometry (Supplementary Figure S1). Thrombocytopenia dose responses reached an average platelet count of 70 × 10^3^ cells/µL when 100% of platelets from isolated PRP were removed, with a less severe but statistically significant impairment in clotting parameters observed on ROTEM (Supplementary Figure S1).

**FIGURE 2:**
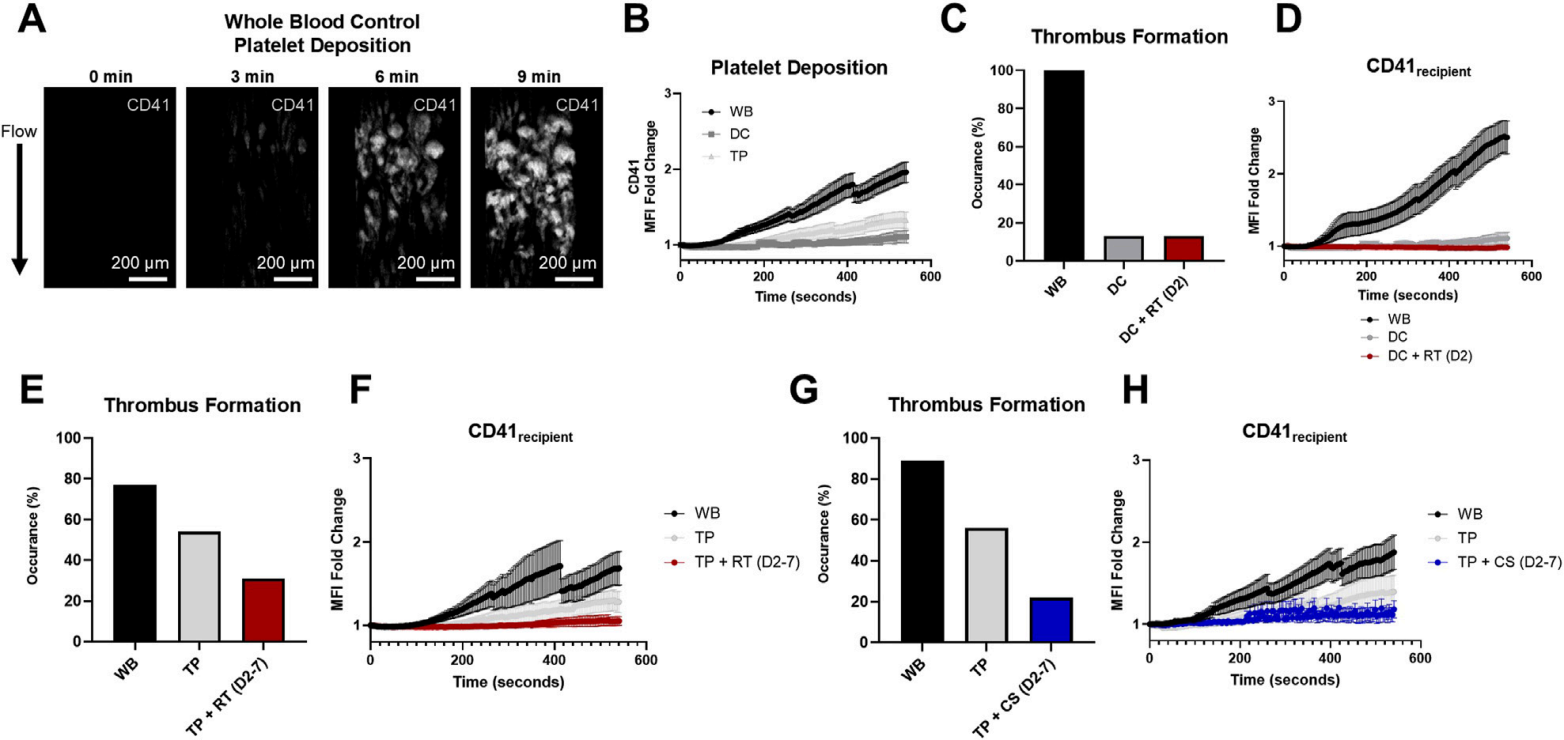
Stenotic microfluidic device assessed *in vitro* hemostatic resuscitation platelet dynamics. **(A)** Representative images of clot formed in the stenotic channel of the microfluidic device (platelets, CD41, white). **(B)** Quantified platelet deposition by the fold change in the CD41 mean fluorescent intensity (MFI) is illustrated in healthy controls (WB) and DC and TP samples. **(C)** Percent occurrence of substantial thrombus formation in the microfluidic device is shown for hemostatic resuscitation experiments in the DC model and **(D)** corresponding platelet deposition quantification of recipient platelets (CD41_recipient_). **(E,G)** Percent occurrence of substantial thrombus formation for hemostatic resuscitation experiments in the thrombocytopenia coagulopathy model and **(F,H)** corresponding platelet deposition quantification of recipient platelets (CD41_recipient_) is shown for RT and CS platelet product mixing conditions. MFI curves are presented as mean ± standard error of the mean.

The stenotic microfluidic device, which showed robust platelet deposition in the stenotic region in whole blood controls (Figure 2A), demonstrated substantial hemostatic dysfunction in DC and TP coagulopathic samples, as measured in fold change of CD41 MFI (Figure 2B). In paired WB and coagulopathy with or without hemostatic resuscitation experiments, RT D2 platelet products did not improve either the occurrence of substantial thrombus formations or recipient platelet deposition (CD41_recipient_) in the DC model (Figures 2C,D). Additionally, although RT products significantly enhanced platelet count recovery upon *in vitro* hemostatic resuscitation in the TP model compared to CS products (Supplementary Figure S2), RT products did not improve thrombus formation occurrence (54% in TP versus 31% in TP + RT (D2-7)) or recipient platelet deposition (CD41_recipient_) (Figures 2E,F) in the TP coagulopathy model. A similar trend was observed with CS products in the TP model, with substantial thrombus formation in 56% of TP versus 22% of TP + CS (D2–7) and no improvement in recipient platelet deposition (CD41_recipient_) compared to coagulopathy (Figures 2G,H).

**FIGURE 3:**
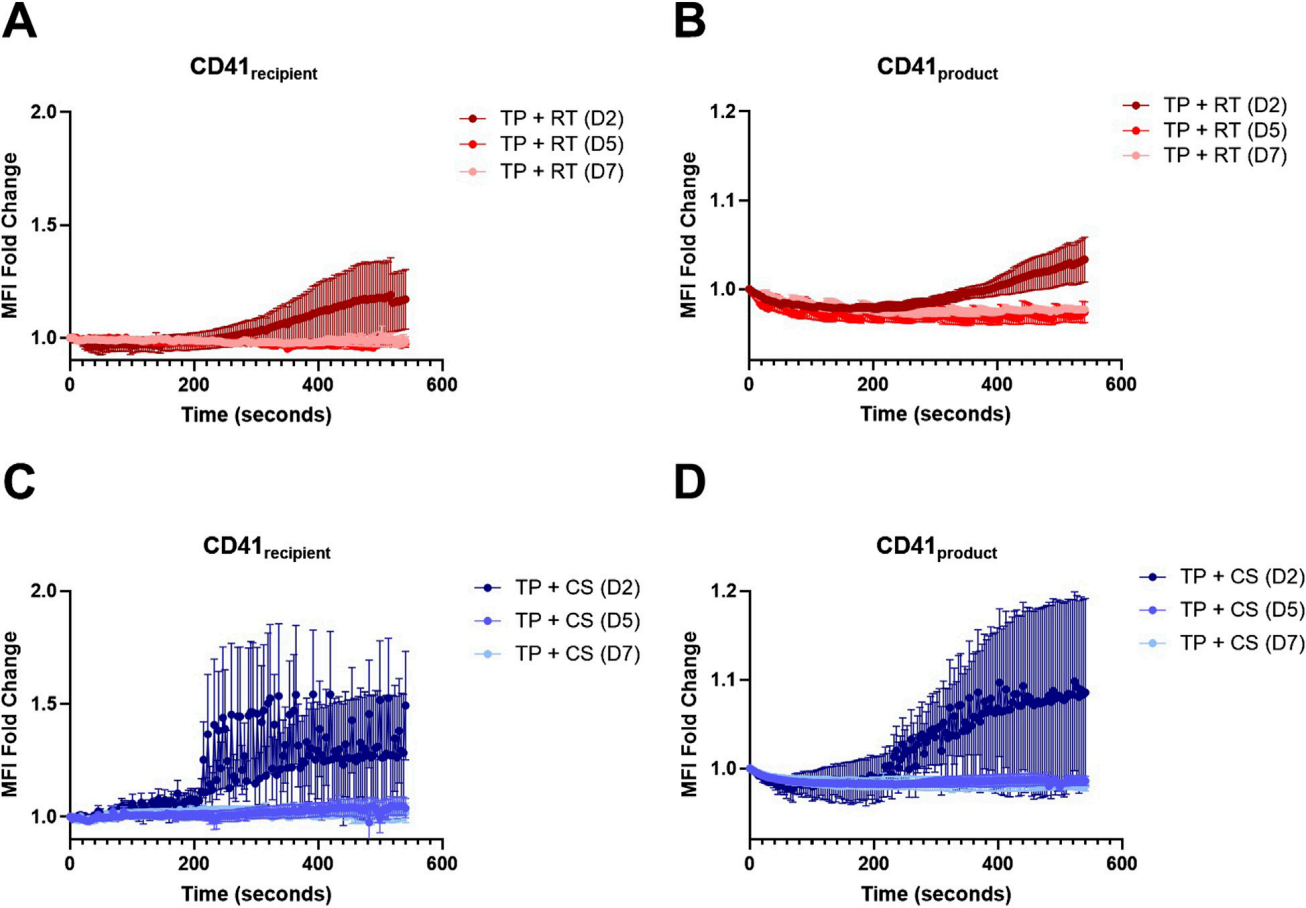
RT and CS platelet dynamics in a stenotic microfluidic device. **(A)** Platelet deposition quantification of recipient platelets (CD41_recipient_) and **(B)** RT platelet products (CD41_product_). **(C)** Platelet deposition quantification of recipient platelets (CD41_recipient_) and **(D)** CS platelet products (CD41_product_). D2, D5, and D7 are shown for both storage conditions. Data are presented as mean ± standard error of the mean.

Platelet deposition of both CD41_recipient_ and CD41_product_ for TP recipient samples mixed with RT and CS platelet products are shown in Figure 3 from stenotic microfluidic experiments. When stratified by storage duration, both RT and CS CD41_product_ were highest at D2, surpassing D5 and D7. Additionally, both RT and CS CD41_recipient_ were highest at D2. Minimal kinetic MFI differences at D5 and D7 were observed.

### 3.3 *In vitro* hemostatic resuscitation in the transected injury microfluidic device

**FIGURE 4:**
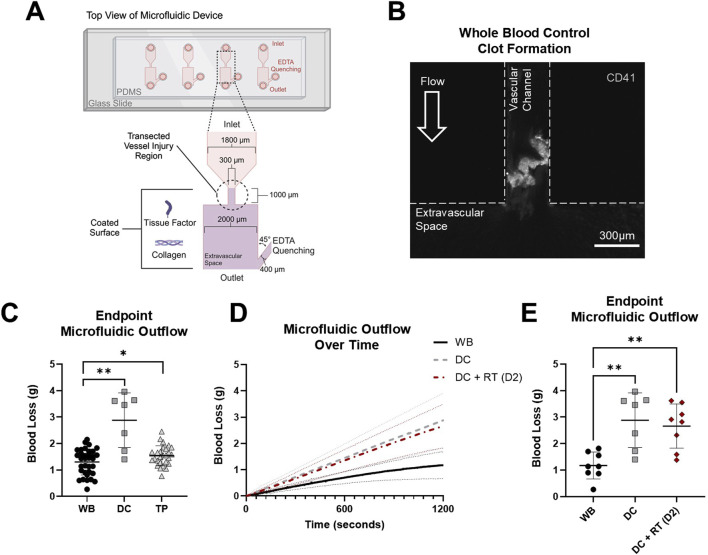
Transected injury microfluidic device assessed blood loss with coagulopathy models to evaluate hemostatic resuscitation. **(A)** Top–down view schematic representation of the transection injury microfluidic device with assembly, dimensions, and coatings used to assess *in vitro* hemostatic resuscitation. **(B)** Representative image of the clot formed in the vascular channel region of the microfluidic device (platelets, CD41, white). **(C)** Blood loss from healthy controls (WB) and DC and TP samples measured via endpoint microfluidic outflow. Data were analyzed via two paired t-tests comparing paired WB controls within each coagulopathy model. *p < 0.05; **p < 0.01. **(D)** Hemostatic resuscitation with RT platelet products at D2 were evaluated with mass outflow curves shown and **(E)** corresponding endpoint microfluidic outflow. Data were analyzed via mixed-effects analysis and Tukey’s post hoc test using a 95% confidence interval. **p < 0.01. All data are presented as mean ± standard deviation or individual values with bars representing mean ± standard deviation.

In the transected microfluidic device (Figure 4A), clots formed in the vascular channel portion of the device with a high platelet content (Figure 4B). WB samples had an average blood loss of 1.301 ± 0.489 g compared to DC with 2.883 ± 1.032 g blood loss (paired samples, p < 0.01) and TP with 1.540 ± 0.374 g blood loss (paired samples, p < 0.05) (Figure 4C). Mass outflow curves (Figure 4D) illustrated reduced flow over 20 min as a clot formed in WB samples. RT platelet products at D2 did not reduce DC blood loss (2.883 ± 1.032 g DC compared to 2.661 ± 0.8362 g DC + RT), and both coagulopathy samples and coagulopathy samples mixed with platelet products had significantly higher blood loss than WB controls (p < 0.01 for both) (Figure 4E).

**FIGURE 5:**
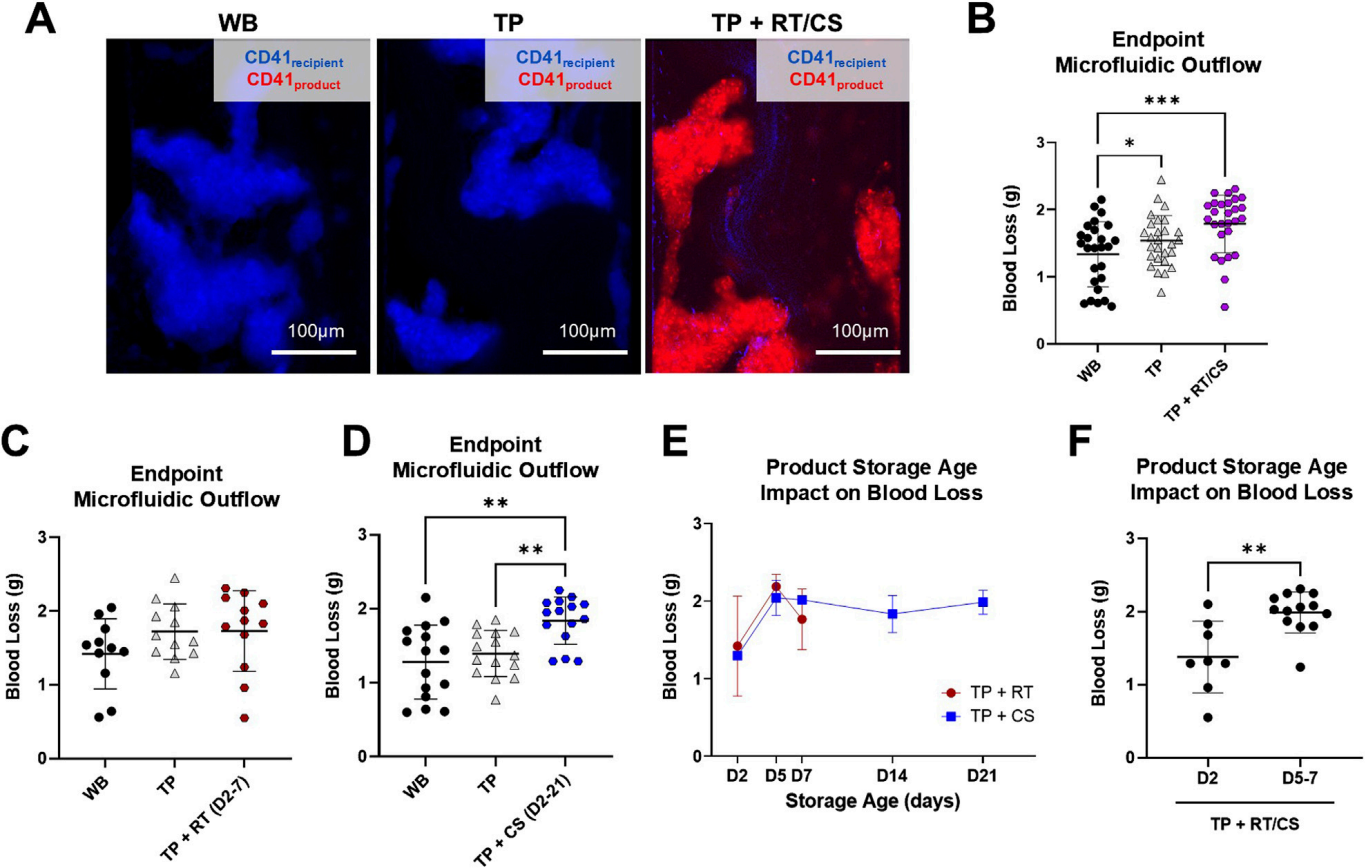
RT and CS products assessed in the thrombocytopenia model in the transected injury microfluidic device. **(A)** Representative images from paired healthy control (WB), TP sample, and TP sample mixed with platelet product are shown. TP sample platelets (CD41_recipient_, blue) and product platelets (CD41_product_, red). **(B)** Blood loss measured via endpoint microfluidic outflow from hemostatic resuscitation experiments combining storage temperature and age. Data were analyzed via mixed-effects analysis and Tukey’s post hoc test using a 95% confidence interval. *p < 0.05; ***p < 0.001. **(C,D)** Blood loss segmented by storage temperature. RT product data were analyzed via mixed-effects analysis and Tukey’s post hoc test using a 95% confidence interval. Cold-storage product data were analyzed via ANOVA and Tukey’s post hoc test using a 95% confidence interval. **p < 0.01. **(E)** Blood loss from TP + RT/CS samples stratified by storage age. **(F)** Blood loss compared in early-storage (D2) versus late-storage (D5 and D7) products. Early- versus late-storage age data were analyzed via an unpaired t-test. **p < 0.01. All data are presented as mean ± standard deviation or individual values, with bars representing mean ± standard deviation.

The ability of platelet products of varying storage age and temperature to elicit a hemostatic effect in a hemostatic resuscitation model was further evaluated in the transected injury microfluidic device with paired donor TP samples. Representative clot images illustrated robust clot formation with recipient platelets (CD41_recipient_, blue) in the microfluidic device that was persistent in the TP model (Figure 5A). Upon mixing platelet products with the TP samples, distinct populations of recipient platelets (CD41_recipient_, blue) and product platelets (CD41_product_, red) were observed in the formed clot. Together, RT and CS platelet products did not reduce TP blood loss (1.540 ± 0.374 g TP compared to 1.790 ± 0.430 g TP + RT/CS), and both the coagulopathy sample and coagulopathy mixed with platelet product had significantly higher blood loss than WB controls (p < 0.05 and p < 0.001, respectively) (Figure 5B). TP + RT platelet product samples, compared to paired coagulopathic TP samples, did not have significantly different blood loss. However, TP + CS platelet product samples had significantly higher blood loss than paired coagulopathic TP samples (1.395 ± 0.312 g TP compared to 1.838 ± 0.320 g TP + CS (D2-21), p < 0.01) (Figures 5C,D). When comparing storage age, D2 products resulted in the lowest blood loss under both storage temperature conditions (1.378 ± 0.492 g with D2 RT and CS products compared to 1.988 ± 0.280 g with D5 and D7 RT and CS products, p < 0.01) (Figures 5E,F).

### 3.4 Recipient platelet and product platelet incorporation following *in vitro* hemostatic resuscitation

**FIGURE 6:**
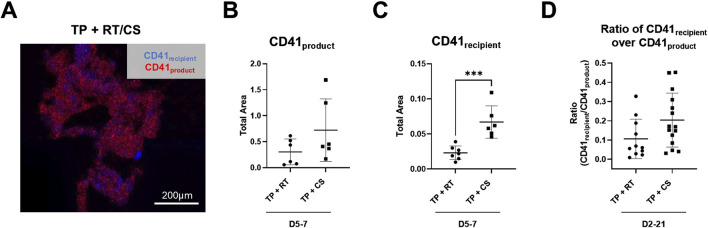
Endpoint thrombus composition in the transected injury microfluidic device. **(A)** Representative image from the TP sample resuscitated with RT platelet products at D2 is shown. TP sample platelets (CD41_recipient_, blue) and product platelets (CD41_product_, red). **(B)** CD41_product_ area quantification and **(C)** CD41_recipient_ area quantification are shown highlighting storage at D5 and D7. **(D)** Calculated ratio of CD41_recipient_ over CD41_product_ area is shown for all storage days. ***p < 0.001. All data are presented as individual values, with bars representing mean ± standard deviation.

Endpoint clot composition in the microfluidic transection model is shown in Figure 6. A representative TP + RT (D2) z-stack image is shown (Figure 6A) with distinct populations from recipient and product platelets observed. When stratified by D5 and D7 where CS products had higher intrinsic hemostatic function on their own, no significant difference in platelet product area (CD41_product_) was observed (Figure 6B). However, a statistically significant increase in recipient platelet area (CD41_recipient_) was observed in CS platelets compared to the RT platelets (p < 0.001), along with an increased ratio of recipient platelets to product platelets across all storage days Figure 6 C, D. Both the recipient and product platelet areas from the transection device and recipient and product platelet MFI endpoints from the stenotic device positively correlated with each other and negatively correlated with transection device blood loss (Supplementary Figure S3).

## 4 Discussion

This study establishes microfluidic platforms that can be used to assess intrinsic product function and hemostatic resuscitation efficacy. In both a stenotic device and a transected injury device, disparate responses with product storage duration were observed. The stenotic device has a simpler design that could be considered closer to a point-of-care possibility, while the transection model is a direct analog to vessel injury in trauma. Further probing of clot composition with respect to recipient and product platelets illustrates disparate responses with product storage temperature.

CS platelets showed increased intrinsic hemostatic function compared to RT platelets, regardless of decreasing platelet count in the product and reduced platelet count recovery in *in vitro* hemostatic resuscitation. This is consistent with a large number of studies in the literature demonstrating increased intrinsic platelet function due to the cold-induced cytoskeletal changes that result in a pro-hemostatic phenotype during CS (Reddoch et al., 2014; Reddoch-Cardenas et al., 2019; Shea et al., 2019; D’Alessandro et al., 2020; George et al., 2023), and specifically, our group (Shea et al., 2024) and others (Montgomery et al., 2013) have shown increased intrinsic product function under flow in CS versus RT platelets. There is little data regarding the direct effects of platelet storage on platelet–endothelial interactions and endothelial function; however, there is evidence that RT platelets can induce the release of soluble mediators from the endothelium and alter its surface marker expression *in vitro*, which increases with storage time (Urner et al., 2012; Baimukanova et al., 2016b; Sut et al., 2018). Some *in vivo* studies also demonstrated the protective effects of RT platelets, which again decrease over the course of storage (Sut et al., 2018). There are very few studies investigating CS platelet–endothelial interactions, although data generated so far show that CS platelets are less effective at preventing endothelial permeability *in vivo* than their RT counterparts (Baimukanova et al., 2016a, p. 22; Miyazawa et al., 2023). Future studies are essential to fully understand the effects of platelet manufacturing approaches and storage conditions on hemostatic, immune, and endothelial function.

The enhancement of *in vitro* hemostatic resuscitation function with CS platelet products was not clearly observed in the stenotic microfluidic device. However, a correlation of the product cell count to the ratio of recipient to product platelet area in the transection device was observed (Supplementary Figure S3). In addition, an enhancement in platelet count recovery, while positively correlated with the product cell count, was also positively correlated with intrinsic product occlusion time (i.e., enhanced platelet count recovery correlates with an increased clotting time or reduced platelet function under flow), supporting the need for further assessment of blood products to accurately evaluate or predict hemostatic function (Supplementary Figure S3). Overall, the direct assessment of blood products and their hemostatic resuscitation efficacy in this study, is a step toward precision transfusion medicine approaches. These data support the consideration of clinical platelet storage policies that prioritize early storage products, when hemostatic activity is at its peak, for trauma patients at risk of developing coagulopathy.

Limitations of this study include a lack of endothelial response. However, our focus was on platelet hemostatic dysfunction, and specifically high-shear hemostatic function under flow, relevant to traumatic injury. Future studies investigating concomitant endothelial and hemostatic effects are warranted, along with the critical roles of other blood cell types, including erythrocytes and immune cells such as neutrophils and monocytes. Additionally, validation of the microfluidic device against *in vivo* clot formation will be investigated in future work. Cold-storage effects on platelet signaling pathways in mixing studies (specifically GPVI and GPIbα) and their implications for recipient hemostasis should also be explored. There is precedent for potential confounding factors in this study, such as platelet donor variability and high variability in assessing function under flow (Fiedler et al., 2020; Shea et al., 2024). Another limitation of this study is a lack of recovery toward healthy control responses upon platelet product mixing in the coagulopathic models examined. Whether this reflects a dilutional effect of product addition to an already coagulopathic blood sample or a deleterious storage lesion effect on hemostatic function remains to be determined.

Pathologically relevant models to assess hemostatic function and resuscitation strategies *in vitro/ex vivo* are critical for high-throughput preclinical investigations. Innovation in this space may ultimately improve outcomes related to trauma and transfusion medicine. A deeper understanding of platelet dysfunction in trauma and the effects of platelet-containing blood products will aid in more accurately modeling coagulopathies in future studies and the interpretation of model outputs. Disparate responses to product storage conditions evaluated in this study may help guide transfusion practices based on functional assessments of blood loss, which were lowest at early storage time points, along with the assessment of recruited recipient platelets in CS platelet products.

## Data Availability

The original contributions presented in the study are included in the article/Supplementary Material, further inquiries can be directed to the corresponding author.

## Supplementary material

SUPPLEMENTARY FIGURE S1 Coagulopathy model dose response. **(A–G)** Dilutional coagulopathy model dose response in citrated blood samples measured via **(A)** complete blood counts for platelet concentration, **(B)** whole blood impedance aggregometry with 10 µM ADP agonist and ROTEM EXTEM **(C)** clotting time, **(D)** clot formation time, **(E)** maximum clot firmness, **(F)** alpha angle, and **(G)** lysis index at 60 min. Dilutions of WB in sterile normal saline were examined from 100% WB and 0% saline to 25% WB and 75% saline. (H–N) Thrombocytopenia model dose response in citrated blood samples measured via **(H)** complete blood counts for platelet concentration, **(I)** whole blood impedance aggregometry with 10 µM ADP agonist and ROTEM EXTEM **(J)** clotting time, **(K)** clot formation time, **(L)** maximum clot firmness, **(M)** alpha angle, and **(N)** lysis index at 60 min. Various centrifugation preparations of PRP (25% of PRP platelets removed to 100% of PRP platelets removed) were conducted for the decreasing platelet count.

SUPPLEMENTARY FIGURE S2 Platelet count recovery. Platelet counts from citrated thrombocytopenia coagulopathy samples before and after simulated transfusion with RT (D2-7) and CS (D2-21) platelet products are shown. Data are presented as individual values with bars representing mean ± standard deviation. Data were analyzed via one-way analysis of variance (ANOVA) and Tukey’s post hoc test using a 95% confidence interval. *p < 0.05; **p < 0.01; ***p < 0.001; ****p < 0.0001.

SUPPLEMENTARY FIGURE S3 Correlation analysis of platelet product assessment and *in vitro* resuscitation. The correlation matrix indicates positive (purple) and negative (orange) Pearson’s correlation coefficient values. *p < 0.05; **p < 0.01; ***p < 0.001.

## References

[B1] Andrews R. K. López JoséA. Berndt M. C. (1997). Molecular mechanisms of platelet adhesion and activation. The Int. J. Biochem. & Cell Biol. 29, 91–105. 10.1016/S1357-2725(96)00122-7 9076944

[B2] Baimukanova G. Miyazawa B. Potter D. R. Gibb S. L. Keating S. Danesh A. (2016a). The effects of 22°C and 4°C storage of platelets on vascular endothelial integrity and function. Transfusion 56 (Suppl. 1), S52–S64. 10.1111/trf.13455 27001362

[B3] Baimukanova G. Miyazawa B. Potter D. R. Muench M. O. Bruhn R. Gibb S. L. (2016b). Platelets regulate vascular endothelial stability: assessing the storage lesion and donor variability of apheresis platelets. Transfusion 56 (Suppl. 1), S65–S75. 10.1111/trf.13532 27001364 PMC5098902

[B41] Berry J. Peaudecerf F. J. Masters N. A. Neeves K. B. Goldstein R. E. Harper M. T. (2021). An “occlusive thrombosis-on-a-chip” microfluidic device for investigating the effect of anti-thrombotic drugs. Lab. Chip. 21 (21), 4104–4117. 10.1039/d1lc00347j 34523623 PMC8547327

[B4] Cap A. P. (2017). Targeting hemorrhage: alternative storage of platelets for hemostatic transfusion. Blood 130 (SCI-32), SCI-32–32. 10.1182/blood.v130.suppl_1.sci-32.sci-32

[B5] Colace T. V. Diamond S. L. (2013). Direct Observation of von Willebrand Factor Elongation and Fiber Formation on Collagen During Acute Whole Blood Exposure to Pathological Flow. Arteriosclerosis, Thrombosis, Vasc. Biol. 33, 105–113. 10.1161/ATVBAHA.112.300522 PMC3595169 23104847

[B6] D’Alessandro A. Thomas K. A. Stefanoni D. Gamboni F. Shea S. M. Reisz J. A. (2020). Metabolic phenotypes of standard and cold‐stored platelets. Transfusion 60, S96–S106. 10.1111/trf.15651 31880330 PMC7971209

[B7] Fiedler S. A. Boller K. Junker A.-C. Kamp C. Hilger A. Schwarz W. (2020). Evaluation of the *in vitro* function of platelet concentrates from pooled buffy coats or apheresis. Transfus. Med. Hemother 47, 314–325. 10.1159/000504917 32884504 PMC7443689

[B8] George C. E. Saunders C. V. Morrison A. Scorer T. Jones S. Dempsey N. C. (2023). Cold stored platelets in the management of bleeding: is it about bioenergetics? Platelets 34, 2188969. 10.1080/09537104.2023.2188969 36922733

[B9] Gunter O. L. Au B. K. Isbell J. M. Mowery N. T. Young P. P. Cotton B. A. (2008). Optimizing outcomes in damage control resuscitation: identifying blood product ratios associated with improved survival. J. Trauma 65, 527–534. 10.1097/TA.0b013e3181826ddf 18784564

[B10] Hegde S. Akbar H. Wellendorf A. M. Nestheide S. Johnson J. F. Zhao X. (2024). Inhibition of RHOA activity preserves the survival and hemostasis function of long-term cold-stored platelets. Blood 144, 1732–1746. 10.1182/blood.2023021453 39088777 PMC11830982

[B11] Hegde S. Akbar H. Zheng Y. Cancelas J. A. (2018). Towards increasing shelf life and haemostatic potency of stored platelet concentrates. Curr. Opin. Hematol. 25, 500–508. 10.1097/MOH.0000000000000456 30281037 PMC6532779

[B12] Hoffmeister K. M. Falet H. Toker A. Barkalow K. L. Stossel T. P. Hartwig J. H. (2001). Mechanisms of cold-induced platelet actin assembly. J. Biol. Chem. 276, 24751–24759. 10.1074/jbc.M011642200 11328807

[B13] Holcomb J. B. Wade C. E. Michalek J. E. Chisholm G. B. Zarzabal L. A. Schreiber M. A. (2008). Increased plasma and platelet to red blood cell ratios improves outcome in 466 massively transfused civilian trauma patients. Ann. Surg. 248, 447–458. 10.1097/SLA.0b013e318185a9ad 18791365

[B14] Johnson L. Tan S. Wood B. Davis A. Marks D. C. (2016). Refrigeration and cryopreservation of platelets differentially affect platelet metabolism and function: a comparison with conventional platelet storage conditions. Transfusion 56, 1807–1818. 10.1111/trf.13630 27158813

[B15] Kushimoto S. Kudo D. Kawazoe Y. (2017). Acute traumatic coagulopathy and trauma-induced coagulopathy: an overview. J. Intensive Care 5, 6. 10.1186/s40560-016-0196-6 34798701 PMC8600738

[B16] Kutcher M. E. Redick B. J. McCreery R. C. Crane I. M. Greenberg M. D. Cachola L. M. (2012). Characterization of platelet dysfunction after trauma. J. Trauma Acute Care Surg. 73, 13–19. 10.1097/TA.0b013e318256deab 22743367 PMC3387387

[B17] Lozano R. Naghavi M. Foreman K. Lim S. Shibuya K. Aboyans V. (2012). Global and regional mortality from 235 causes of death for 20 age groups in 1990 and 2010: a systematic analysis for the Global Burden of Disease Study 2010. Lancet 380, 2095–2128. 10.1016/S0140-6736(12)61728-0 23245604 PMC10790329

[B18] Mihalko E. Abdullah A. Hoteit L. Akinbode D. Shea S. Neal M. D. (2024). Microfluidics in assessing platelet function. J. Vis. Exp. (JoVE), e67214. 10.3791/67214 PMC12336984 39584679

[B19] Miyazawa B. Trivedi A. Vivona L. Lin M. Potter D. Nair A. (2023). Histone deacetylase-6 modulates the effects of 4°C platelets on vascular endothelial permeability. Blood Adv. 7, 1241–1257. 10.1182/bloodadvances.2022007409 36375044 PMC10090218

[B20] Montgomery R. K. Reddoch K. M. Evani S. J. Cap A. P. Ramasubramanian A. K. (2013). Enhanced shear-induced platelet aggregation due to low-temperature storage. Transfusion 53, 1520–1530. 10.1111/j.1537-2995.2012.03917.x 23043289 PMC4321779

[B21] Moore E. E. Moore H. B. Kornblith L. Z. Neal M. D. Hoffman M. Mutch N. J. (2021). Trauma-induced coagulopathy. Nat. Rev. Dis. Prim. 7, 30–23. 10.1038/s41572-021-00264-3 33927200 PMC9107773

[B22] Ng M. S. Y. Tung J.-P. Fraser J. F. (2018). Platelet storage lesions: what more do we know now? Transfus. Med. Rev. S0887-7963 (17), 144–154. 10.1016/j.tmrv.2018.04.001 29751949

[B23] Reddoch K. M. Pidcoke H. F. Montgomery R. K. Fedyk C. G. Aden J. K. Ramasubramanian A. K. (2014). Hemostatic function of apheresis platelets stored at 4°C and 22°C. Shock 41, 54–61. 10.1097/SHK.0000000000000082 24169210 PMC3991734

[B24] Reddoch-Cardenas K. M. Bynum J. A. Meledeo M. A. Nair P. M. Wu X. Darlington D. N. (2019). Cold-stored platelets: a product with function optimized for hemorrhage control. Transfus. Apher. Sci. 58, 16–22. 10.1016/j.transci.2018.12.012 30704925

[B25] Shankaran H. Alexandridis P. Neelamegham S. (2003). Aspects of hydrodynamic shear regulating shear-induced platelet activation and self-association of von Willebrand factor in suspension. Blood 101, 2637–2645. 10.1182/blood-2002-05-1550 12456504

[B26] Shea S. M. Reisz J. A. Mihalko E. P. Rahn K. C. Rassam R. M. G. Chitrakar A. (2024). Cold-stored platelet hemostatic capacity is maintained for three weeks of storage and associated with taurine metabolism. J. Thromb. Haemost. 22, 1154–1166. 10.1016/j.jtha.2023.11.025 38072374

[B27] Shea S. M. Spinella P. C. Thomas K. A. (2022). Cold-stored platelet function is not significantly altered by agitation or manual mixing. Transfusion 62, 1850–1859. 10.1111/trf.17005 35898113

[B28] Shea S. M. Thomas K. A. Spinella P. C. (2019). The effect of platelet storage temperature on haemostatic, immune, and endothelial function: potential for personalised medicine. Blood Transfus. 17, 321–330. 10.2450/2019.0095-19 31385802 PMC6683864

[B29] Sloos P. H. Vulliamy P. van ’t Veer C. Gupta A. S. Neal M. D. Brohi K. (2022). Platelet dysfunction after trauma: from mechanisms to targeted treatment. Transfusion 62, S281–S300. 10.1111/trf.16971 35748694 PMC9546174

[B30] Sperry J. L. Guyette F. X. Brown J. B. Yazer M. H. Triulzi D. J. Early-Young B. J. (2018). Prehospital plasma during air medical transport in trauma patients at risk for hemorrhagic shock. N. Engl. J. Med. 379, 315–326. 10.1056/NEJMoa1802345 30044935

[B31] Sut C. Hamzeh-Cognasse H. Arthaud C.-A. Eyraud M.-A. Chettab K. Dumontet C. (2018). Platelet concentrate supernatants alter endothelial cell mRNA and protein expression patterns as a function of storage length. Transfusion 58, 2635–2644. 10.1111/trf.14973 30325037

[B32] Torres C. M. Kenzik K. M. Saillant N. N. Scantling D. R. Sanchez S. E. Brahmbhatt T. S. (2024). Timing to first whole blood transfusion and survival following severe hemorrhage in trauma patients. JAMA Surg. 159, 374–381. 10.1001/jamasurg.2023.7178 38294820 PMC10831629

[B33] Triulzi D. J. Kickler T. S. Braine H. G. (1992). Detection and significance of alpha granule membrane protein 140 expression on platelets collected by apheresis. Transfusion 32, 529–533. 10.1046/j.1537-2995.1992.32692367196.x 1380189

[B34] Urner M. Herrmann I. K. Buddeberg F. Schuppli C. Roth Z’graggen B. Hasler M. (2012). Effects of blood products on inflammatory response in endothelial cells *in vitro* . PLoS One 7, e33403. 10.1371/journal.pone.0033403 22438924 PMC3306413

[B35] Vulliamy P. Kornblith L. Z. Kutcher M. E. Cohen M. J. Brohi K. Neal M. D. (2021). Alterations in platelet behavior after major trauma: adaptive or maladaptive? Platelets 32, 295–304. 10.1080/09537104.2020.1718633 31986948 PMC7382983

[B36] Vulliamy P. Montague S. J. Gillespie S. Chan M. V. Coupland L. A. Andrews R. K. (2020). Loss of GPVI and GPIbα contributes to trauma-induced platelet dysfunction in severely injured patients. Blood Adv. 4, 2623–2630. 10.1182/bloodadvances.2020001776 32556282 PMC7322946

[B37] Winokur R. Hartwig J. H. (1995). Mechanism of shape change in chilled human platelets. Blood 85, 1796–1804. 10.1182/blood.v85.7.1796.bloodjournal8571796 7703486

[B38] Wood B. Padula M. P. Marks D. C. Johnson L. (2016). Refrigerated storage of platelets initiates changes in platelet surface marker expression and localization of intracellular proteins. Transfusion 56, 2548–2559. 10.1111/trf.13723 27460096

[B39] Yakusheva A. A. Butov K. R. Bykov G. A. Závodszky G. Eckly A. Ataullakhanov F. I. (2022). Traumatic vessel injuries initiating hemostasis generate high shear conditions. Blood Adv. 6, 4834–4846. 10.1182/bloodadvances.2022007550 35728058 PMC9631664

[B40] Zhang Y. Ehrlich S. M. Zhu C. Du X. (2022). Signaling mechanisms of the platelet glycoprotein Ib-IX complex. Platelets 33, 823–832. 10.1080/09537104.2022.2071852 35615944 PMC9378482

