# Supplementary figures and images for: Microfluidic transection injury and high-shear thrombus formation demonstrate increased hemostatic efficacy of cold-stored platelets and in vitro resuscitation in induced coagulopathy models
Source: Front Bioeng Biotechnol. 2025 May 12;13:1568113. doi: 10.3389/fbioe.2025.1568113 (PMC12104287; doi:10.3389/fbioe.2025.1568113)

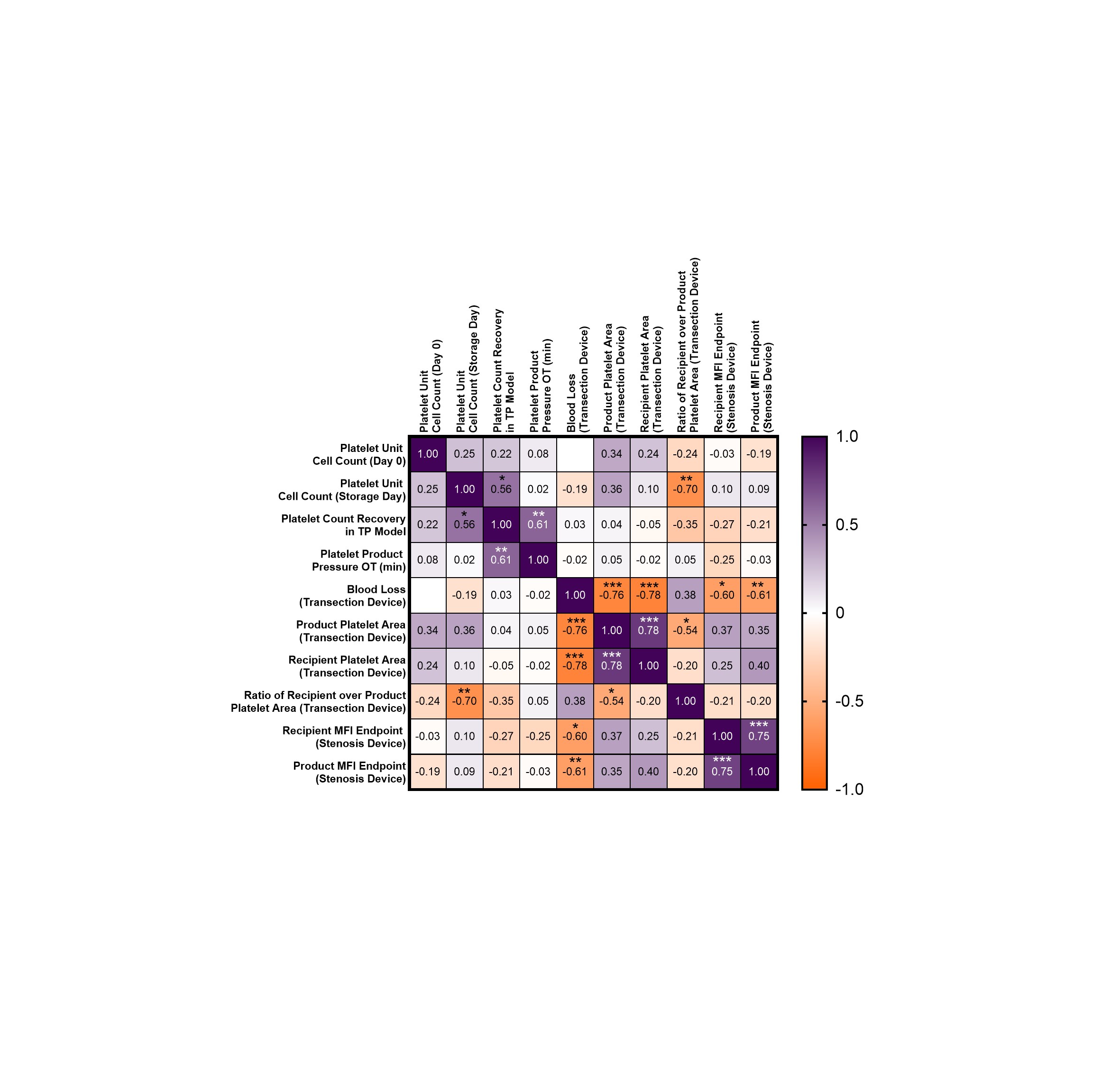

Supplement: Supplementary file 1 [file Image3.jpeg]

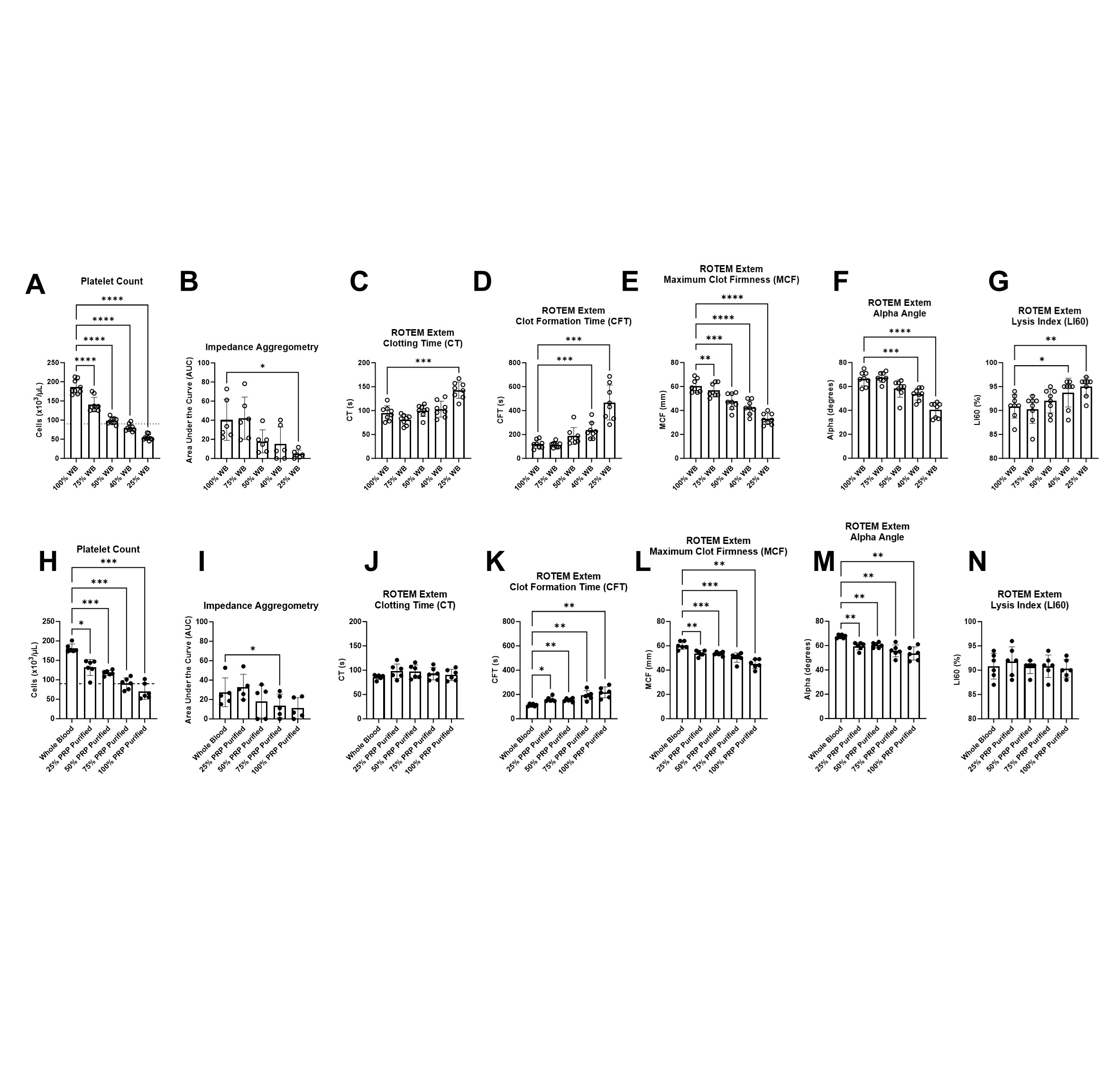

Supplement: Supplementary file 3 [file Image1.jpeg]

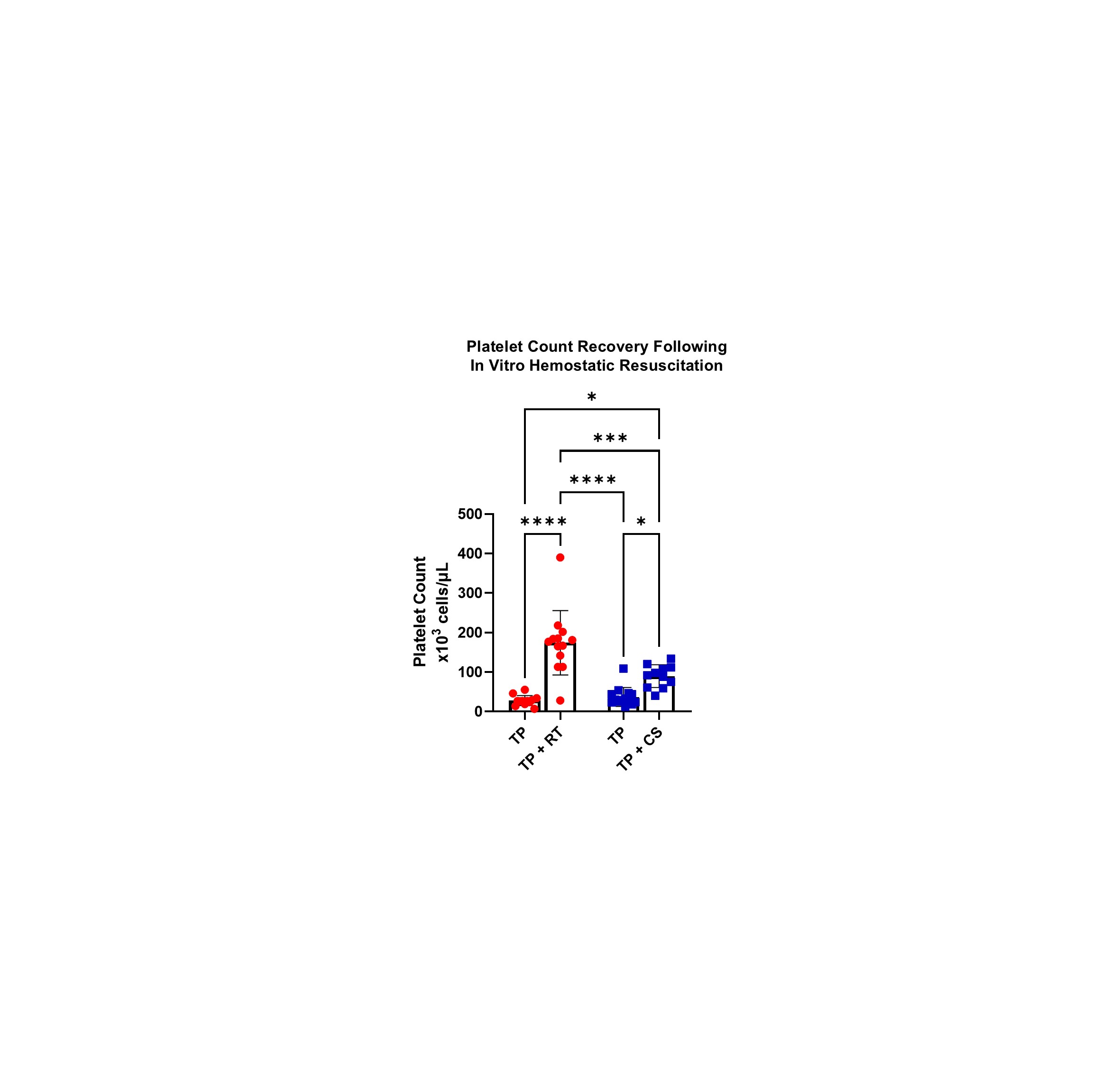

Supplement: Supplementary file 4 [file Image2.jpeg]
