# Supplementary material for: Microfluidic transection injury and high-shear thrombus formation demonstrate increased hemostatic efficacy of cold-stored platelets and in vitro resuscitation in induced coagulopathy models
Source: Front Bioeng Biotechnol. 2025 May 12;13:1568113. doi: 10.3389/fbioe.2025.1568113 (PMC12104287; doi:10.3389/fbioe.2025.1568113)
Supplement: Supplementary file 2 [file Table1.docx]

**Supplemental Material: Inclusion and Exclusion Criteria for Human Subjects**

Whole Blood Donors (IRB: 21100141):

Inclusion Criteria:

1. Not currently feeling sick or unwell
2. Age >/= 18 years of age
3. Weight >/= 110 lbs (50kg)
4. Not pregnant

Exclusion Criteria:

1. Age <18 years of age
2. Weight < 110 lbs
3. Sick or unwell
4. Pregnant

Apheresis Donors (IRB: 21110093):

Inclusion Criteria:

1. Not currently feeling sick or unwell
2. All ABO Blood Types
3. Age >/= 18 years of age
4. Weight >/= 110 lbs (50kg)
5. Body Mass Index (BMI) between 18.5 - 35 kg/m^2^
6. Normal vital signs prior to blood draw (standard blood collection center criteria for apheresis platelet donation)
   1. Pulse between 50 - 100 beats/min and regular
   2. Temperature between 96.0°F - 99.5°F (35.6°C - 38.6°C)
   3. Systolic blood pressure range between 90 - 180 mmHg
   4. Diastolic blood pressure range between 50 - 100 mmHg
7. Hemoglobin level between 12.5 – 20 g/dL
8. The venipuncture site should be free of any lesion or scar of needle pricks indicative of addiction to narcotics or frequent blood donation (as in the case of professional blood donors)
9. In the opinion of the investigator, absence of any significant medical history, physical abnormalities, addictive diseases, or underlying medical conditions that may preclude the subject from being a good study candidate
10. Written informed consent to participate in the study provided by the subject
11. Hematocrit level between 36 – 55 %
12. Platelet level between 150 - 400 x10^3^ cells/µL

Exclusion Criteria:

1. Current daily tobacco use (including smokeless tobacco)
2. Pregnant or lactating at the time of study
3. Currently taking (within the past 7 days) any medications to affect coagulation, such as:
   1. Anticoagulants (e.g. Coumadin/Warfarin, Heparin, Enoxaparin, direct thrombin inhibitors, direct anti-Xa inhibitors)
   2. Aspirin
   3. Antiplatelet drugs (e.g.Ticlopidine/Ticlid, Clopidogrel/Plavix)
4. History of excessive bleeding from minor trauma that required medical attention
5. History of blood related disease (e.g. anemia, sickle cell disease)
6. History of any congenital or acquired coagulopathies (e.g. hemophilia)
7. History of any thromboembolic diseases (e.g. deep vein thrombosis (DVT), pulmonary embolism)
8. History of HIV or hepatitis
9. Donated blood, platelets, or plasma within the past 1 week
10. Received a blood transfusion in the last 3 months
11. Donated a double unit of red cells using an apheresis machine in the past 16 weeks
12. Currently involved in another clinical study where that participation may conflict or interfere with the diagnostic results of this clinical study
